# Robotic-assisted proximal gastrectomy using the double-flap technique for early gastric cancer with situs inversus totalis: a case report

**DOI:** 10.1186/s40792-021-01262-z

**Published:** 2021-08-02

**Authors:** Atsushi Takeno, Toru Masuzawa, Shinsuke Katsuyama, Kohei Murakami, Kenji Kawai, Yoshiteru Katsura, Yoshiaki Ohmura, Yoshinori Kagawa, Yutaka Takeda, Taishi Hata, Kohei Murata

**Affiliations:** 1 Department of Gastrointestinal Surgery, Kansai Rosai Hospital, Amagasaki, Japan; 2 Department of Surgery, National Hospital Organization Osaka National Hospital, 2-1-14, Hoenzaka, Chuoku, Osaka, 540-0006 Japan; 3 Department of Surgery, Japan Community Heath Care Organization Osaka Hospital, Osaka, Japan

**Keywords:** Gastrectomy, Robotic surgical procedures, Situs inversus

## Abstract

**Background:**

The robotic system has been applied in the treatment of gastric cancer (GC), and the procedure has been found to be safe and feasible. Situs inversus totalis (SIT) is a relatively rare autosomal recessive congenital anomaly. We successfully performed robot-assisted proximal gastrectomy (RAPG) and handsewn double-flap esophagogastrostomy for GC in a patient with SIT.

**Case presentation:**

A 71-year-old woman was referred to us with an asymptomatic ulcerative lesion in the upper body of the stomach. Computed tomography revealed that she had SIT. She was diagnosed with cT1bN0M0, cStageIA gastric cancer. RAPG with lymph node dissection and handsewn double-flap esophagogastrostomy was performed. Robotic surgery enabled the surgeon to perform the surgery without changing his position and experiencing any confusion resulting from the patient’s reversed anatomy. It took 448 min, and no intraoperative complications occurred. Her postoperative course was uneventful; she was discharged on postoperative day 10. The final pathologic report showed pT1b1N0M0, pStage IA.

**Conclusions:**

This is the first case describing RAPG with handsewn double-flap esophagogastrostomy for a SIT patient with early GC.

## Background

Situs inversus totalis (SIT) is a relatively rare autosomal recessive congenital anomaly found in 1 per 4000–8000 persons. It is characterized by reverse positioning of the major visceral organs from their usual positions. Laparoscopic gastrectomy (LG) in SIT patients with gastric cancer (GC) was first reported in 2003, and standard typical lymph node dissection has been reported recently in 2010 [1,2].

The first case of robot-assisted gastrectomy (RG) in SIT patients with GC was reported in 2012 [3]. Kim mentioned that RG was more suitable than LG, because the surgeon did not have to change his position and could change hand easily. Five SIT cases with GC who underwent RG have been reported (3 DG and 2 TG cases) in the past [4–7]. However, to the best of our knowledge, there are no reports of robotic-assisted proximal gastrectomy (RAPG) for upper-third GC in the literature.

The double-flap esophagogastrostomy technique (DFT) has been applied to laparoscopic PG to prevent reflex. In spite of effective prevention of reflex, it is technically demanding because of complicated suturing and ligation maneuvers. Shibasaki reported that RG was more advantageous to perform intracorporeal hand-sewing anastomosis than LG, because robotic assistance could allow to master it with a short learning curve [8]. We describe a case of RAPG with lymph node dissection and handsewn DFT for upper-third early GC (EGC).

## Case presentation

**Fig. 1:**
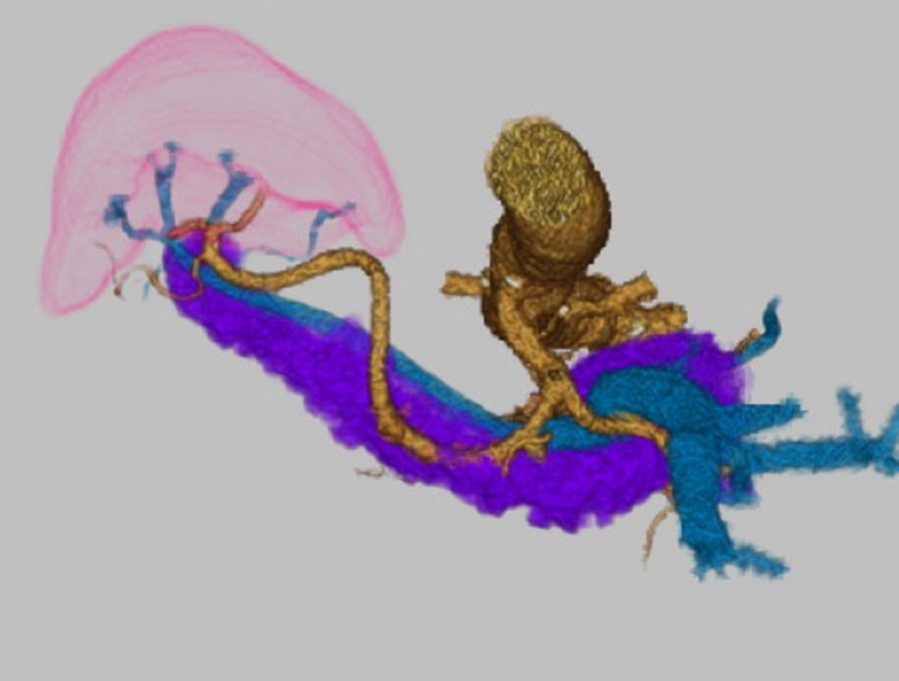
Three-dimensional reconstruction of an abdominal computed tomography angiogram preoperatively

An asymptomatic ulcerative lesion was found during screening gastroscopy of the upper body of the stomach in a 71-year-old woman. On the endoscopic ultrasonogram, the 2-cm ulcerative lesion was invading the submucosal layer. Computed tomography (CT) revealed that she had SIT without lymph node or distant metastases, and three-dimensional (3D) reconstruction of an abdominal CT angiogram showed no vessel anomalies (Fig. 1). We diagnosed her with EGC, U, post, cType 0–IIc, cT1bN0M0, cStageIA, and it was thought to be contraindicated for endoscopic resection. We decided to perform robotic-assisted PG (using da Vinci Xi Surgical Systems) and D1 + lymphadenectomy based on Japanese gastric cancer treatment guidelines 2018 (5th edition) [9].

**Fig. 2:**
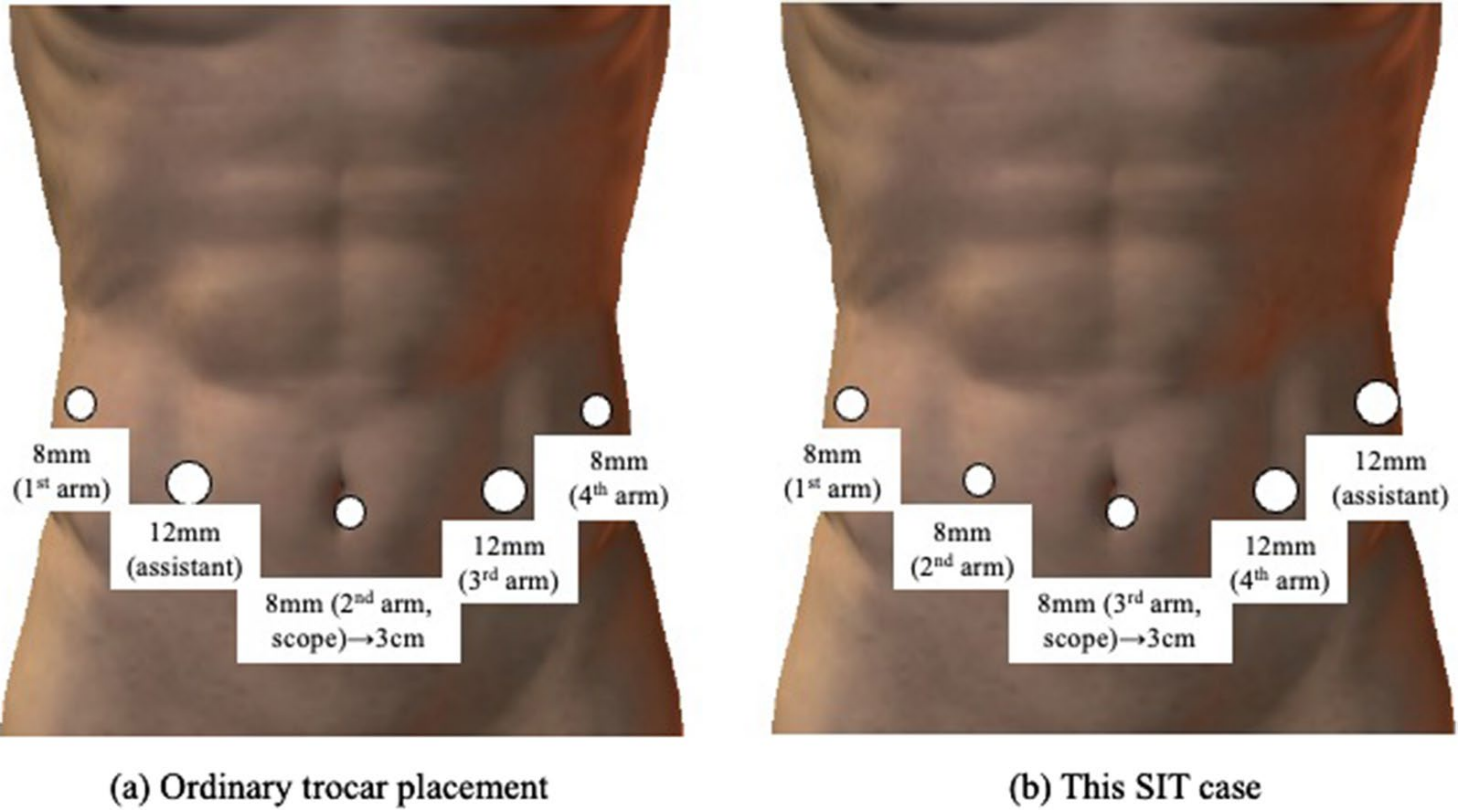
Placement of the trocars and robotic arms. **a** Ordinary trocar placement. **b** This SIT case

A scope was inserted into the abdominal cavity through an 8-mm port on the umbilicus, and four more trocars were inserted. As shown in Fig. 2, the location of the trocar placement is the same as usual, whereas we adjusted their arrangement. In performing RAG, we normally set 4th arm at the left lateral side to develop macro-surgical field and handle 1st arm at the right lateral and 3rd arm at the left medial to proceed the surgical procedure. In this SIT case, we adjusted to set 1st arm at the right lateral as developing the field and handle 2nd at the left medial and 4th at the left medial, because it might be difficult to approach to the esophagus and cardia located in the right upper area if we had adopted normal setting. Accordingly, assistant trocar was moved from the right medial to the left lateral.

The liver was retracted with internal organ retractors. After a thorough examination, the greater omentum was divided 3 cm away from the gastroepiploic vessels. The right (left in the normal anatomy) gastroepiploic artery and vein were clipped and divided near the spleen. The gastrosplenic ligament was divided by a sealing device. Next, the lesser omentum was opened and the lymph nodes along the lesser curvature of the stomach were dissected. We moved to the suprapancreatic area, and lymph node numbers 8a, 9, and 7 were dissected safely. Finally, by tracing the splenic artery behind the splenic vein, the number 11 group of lymphatic tissue was harvested. The postperitoneal fascia between the upper border of the pancreatic tail and cardia was dissected along a plane superior to the Gerota fascia. The pericardiac and periesophageal tissue in the esophageal hiatus was dissected, and the esophagus was transected with the Endowrist Stapler. The stomach was extracted through a 3-cm umbilical incision and resected at the level of the upper one-third of the stomach.

**Fig. 3:**
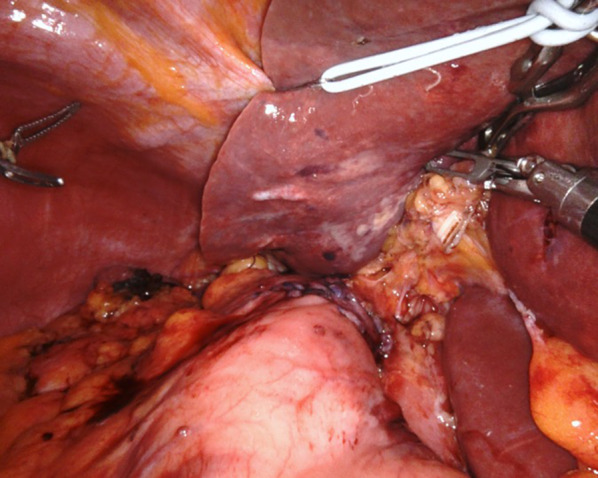
Intraoperative findings after esophagogastrostomy

The double seromuscular flaps were prepared extracorporeally at the anterior wall of the remnant stomach in order to cautiously remove the submucosal layer from the mucosal layer. After establishing the pneumoperitoneum again, we performed handsewn esophagogastrostomy intracorporeally. Firstly, four stitches were used to fix the posterior wall of the esophagus to the superior edge of the mucosal window. Secondly, the posterior wall of the esophagus and the superior opening of the mucosa on the remnant stomach were closed by continuous suturing using barbed sutures. The anterior wall of the esophagus and gastric wall at the lower end of the flap were also anastomosed layer-by-layer by continuous suturing using barbed sutures. Finally, the anastomosis was finished by covering the anastomosis site with seromuscular flaps using barbed sutures. An air leakage test was performed to confirm closure of the anastomosis (Fig. 3).

The operative time was 448 min, and blood loss was 45 ml. The final pathology demonstrated a poorly differentiated 0–IIc lesion with invasion to the submucosa. There was no metastasis in any of the retrieved lymph nodes. The final stage was pT1b1N0M0, pStage IA according to the Japanese Classification of Gastric Carcinoma staging system. No intraoperative complications occurred. The patient’s postoperative course was uneventful; she was discharged on postoperative day 10.

## Discussion

SIT is a rare congenital anomaly. SIT can be accompanied by cardiopulmonary malformations, familial long QT syndrome, total esophageal duplication, agnathia, and various urologic anomalies [10]. Therefore, it is important to identify any abnormal vasculature preoperatively, because it is associated with a risk of misidentifying anatomy and causing unanticipated injury of important vessels. Our patient had no major vascular abnormalities. However, it is necessary to examine 3D reconstruction of a blood stream from an abdominal CT angiogram preoperatively, as shown in Fig. 1.

RG for GC has been developed for minimally invasive surgery and is thought to have the potential to overcome the shortcomings of LG. A comparative study demonstrated that RG is as acceptable as LG in surgical and oncologic outcomes with lower estimated blood loss, acceptable complications, and radical resection [11]. A recent study about long-term oncologic outcomes of RG and LG revealed that RG is an oncological alternative [12].

Only small cases of GC have been reported in patients with SIT to date. Of these, only five patients underwent RG, 3 for DG [3,^5^,7] and the other 2 for TG [4,6]. Additionally, nine patients underwent laparoscopic-assisted DG and only one patient underwent TG [10]. In laparoscopic surgery, little ingenuity in moving the monitor and standing opposite the usual side are required. However, in robotic surgery like in our case, the surgeon could perform the operation without changing his position and experiencing any confusion resulting from the patient’s reversed anatomy. Indeed, we successfully completed RAPG in the same procedure as normal cases besides adjusting the trocar arrangement.

We adopted the double-flap esophagogastrostomy technique (DFT) after PG. This technique was developed to prevent reflux after PG has been applied to laparoscopic proximal gastrectomy (LPG-DFT) [13]. It is achieved by burying the abdominal esophagus into the gastric submucosa. It has been reported to be a better surgical procedure for treating upper-third EGC than laparoscopic TG in morbidity, postoperative hospital stay, and postoperative nutritional status [14]. Kuroda also reported that it was a feasible option for effective prevention of reflex based on the results of multicenter retrospective study [15]. However, LPG-DFT is technically demanding because of complicated suturing and ligation maneuvers. Robotic assistance may be useful for valvuloplastic esophagogastrostomy-DFT with a short learning curve [8].

## Conclusion

This is the first case describing RAPG with handsewn double-flap esophagogastrostomy for a SIT patient with early GC. RG may be suitable to perform proximal gastrectomy for such a complicated SIT case because it can resolve technical problems.

## Data Availability

Data sharing is not applicable to this article as no datasets were generated or analyzed during the current study.
